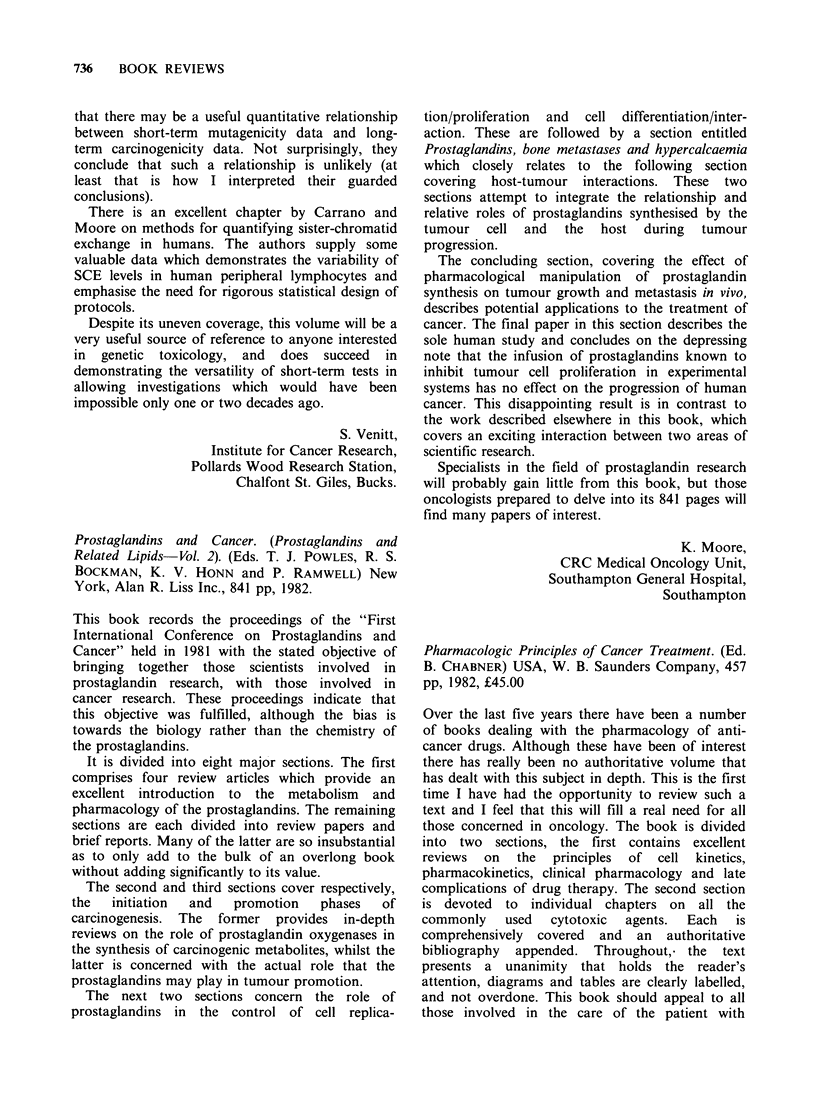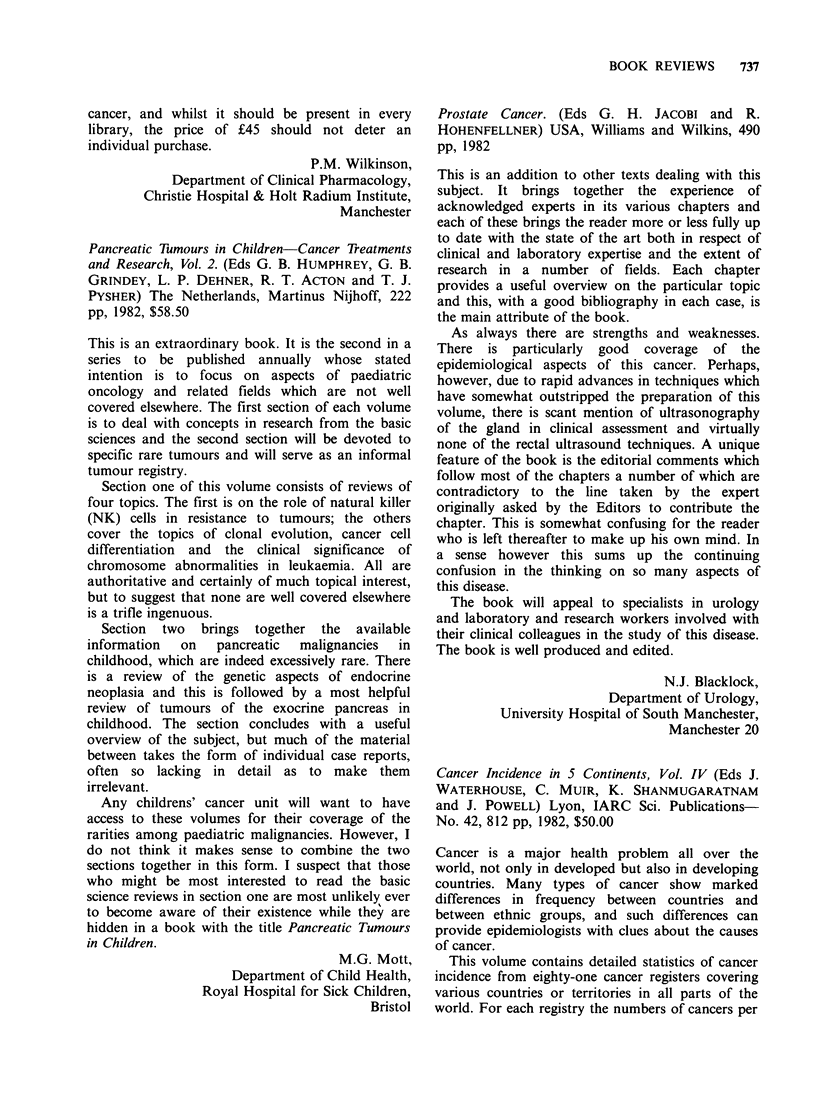# Pharmacologic Principles of Cancer Treatment

**Published:** 1983-05

**Authors:** P.M. Wilkinson


*Pharmacologic Principles of Cancer Treatment.* (Ed. B. CHABNER) USA, W. B. Saunders Company, 457 pp, 1982, £45.00

Over the last five years there have been a number of books dealing with the pharmacology of anti-cancer drugs. Although these have been of interest there has really been no authoritative volume that has dealt with this subject in depth. This is the first time I have had the opportunity to review such a text and I feel that this will fill a real need for all those concerned in oncology. The book is divided into two sections, the first contains excellent reviews on the principles of cell kinetics, pharmacokinetics, clinical pharmacology and late complications of drug therapy. The second section is devoted to individual chapters on all the commonly used cytotoxic agents. Each is comprehensively covered and an authoritative bibliography appended. Throughout,· the text presents a unanimity that holds the reader's attention, diagrams and tables are clearly labelled, and not overdone. This book should appeal to all those involved in the care of the patient with cancer, and whilst it should be present in every library, the price of £45 should not deter an individual purchase.

P.M. Wilkinson,
Department of Clinical Pharmacology,
Christie Hospital & Holt Radium Institute,
Manchester